# The efficacy and safety of pyrotinib in treating HER2‐positive breast cancer patients with brain metastasis: A multicenter study

**DOI:** 10.1002/cam4.4481

**Published:** 2021-12-27

**Authors:** Min Gao, Chao Fu, Shanshan Li, Fang Chen, Yongteng Yang, Chunjian Wang, Jie Qin, Shuaishuai Liu, Ranran Zhang, Changyuan Wang, Jinbao Zong, liping Meng, Xiangjiao Meng

**Affiliations:** ^1^ Department of Radiation Therapy, Shandong Cancer Hospital and Institute Shandong First Medical University and Shandong Academy of Medical Sciences Jinan China; ^2^ Chinese Medicine Hospital of Gaomi Gaomi China; ^3^ Zibo Municipal Hospital Zibo China; ^4^ Department of Ultrasonography the Second Affiliated Hospital of Shandong University of Traditional Chinese Medicine Jinan China; ^5^ First People’s Hospital of Ning Yang Ningyang China; ^6^ Breast Cancer Center, Shandong Cancer Hospital and Institute Shandong First Medical University and Shandong of Medical Sciences Jinan Shandong China; ^7^ Zibo Gaoqing people's Hospital Zibo China; ^8^ Zao zhuang Municipal Hospital Zaozhuang China; ^9^ XueCheng Distict People Hospital Zaozhuang China; ^10^ Department of Dermatology Qingdao Municipal Hospital Group Qingdao China; ^11^ Qingdao Hospital of Traditional Chinese Medicine The affiliated Qingdao Hiser Hospital of Qingdao University Qingdao China; ^12^ The third people's Hospital of Dezhou Dezhou China

**Keywords:** efficacy, HER2‐positive breast cancers with brain metastasis, pyrotinib, radiotherapy, safety

## Abstract

**Purpose:**

To investigate the efficacy and safety of pyrotinib in treating patients with human epidermal growth factor receptor type 2 (HER2)‐positive breast cancers with brain metastasis.

**Patients and Methods:**

This is a multicenter retrospective study, and the HER2‐positive breast cancer patients with brain metastasis were studied. The enrolled patients were given pyrotinib 400 mg orally once per day for 21 days as one cycle, and evaluated every two cycles. All relevant data were detected for final assessments including medical history, clinical examination, histopathology, immunohistochemistry, radiographic imaging, treatment outcome, and adverse events.

**Results:**

Forty‐two female patients in total were enrolled in this study. The objective response rate (ORR) and disease control rate (DCR) of central nervous system (CNS), were found in 20 of 42 (47.6%) and in 39 of 42 (92.8%), respectively, while for extra‐CNS, the respective ORR and DCR were in 9 of 38 (23.6%) and in 36 of 38 (94.7%), respectively. The compounded ORR and DCR were seen in 17 of 42 (40.4%) and in 39 of 42 (92.8%), respectively. The improvement rate of craniocerebral symptoms after treatment was (19/19) 100% and the median duration was 15 months. The median effective time of brain metastases and other metastases was 43 and 50 days. The median follow‐up time was 22 months (interquartile range, 16.0–24.3 months). The median time for progression in brain metastasis was 16.6 months. The median time to progress for our group patients was 11.1 months. Sixteen patients (36%) with adverse reactions were recorded in the study.

**Conclusion:**

Pyrotinib combined with chemotherapy/radiotherapy or alone showed significantly greater local control rates and progression free survival (PFS), with manageable toxicity for patients with HER2‐positive breast cancer with brain metastases, and further follow‐up will provide an overall survival (OS) data.

## Graphical abstract

Currently, there is less data of pyrotinib in treatment HER2‐positive breast cancer patients with brain metastases, and the efficacy and safety of combined radiotherapy is unknown. Our study show that pyrotinib alone led to significantly greater local control rates and PFS, with manageable toxicity for patients with HER2‐positive breast cancer with brain metastases. Further follow‐up study will provide more OS data.

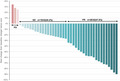

## 1 INTRODUCTION

Breast cancer (BC) is the most frequent female cancer and the primary cause of cancer‐related death in women worldwide.^1^, ^2^ The activation of human epidermal growth factor receptor type 2 (HER2) by amplification and protein overexpression^3^, ^4^ was reported in 14.5%–15% breast cancers. Compared to the primary cancer, higher association of brain metastasis with HER2 activation has been demonstrated.^5^, ^6^, ^7^

In the case of solitary brain metastases, surgery or stereotactic radiosurgery is the preferred therapeutic approaches. Stereotactic radiosurgery is an option for patients with up to three brain metastases. In the case with diffuse brain metastasis or present with poor performance status, whole‐brain radiation therapy (WBRT) is the standard of care.^8^

The HER2‐targeted therapies, including trastuzumab, pertuzumab, lapatinib, and ado‐trastuzumab emtansine, have been developed and investigated during the past two decades, which definitely improved the treatment efficacy in patients with HER2‐positive breast cancer.^9^, ^10^, ^11^, ^12^ However, there are few reports to guide the treatment of HER2‐positive breast cancer patients with brain metastasis, and the treatment resistance to anti‐HER2 therapy as well as its occasional intolerable adverse reactions remains to be a challenge, highlighting a clear urgent demand for more novel therapies.^13^

Pyrotinib is an oral, irreversible pan‐ErbB receptor tyrosine kinase inhibitor (TKI) with activity against epidermal growth factor receptor (EGFR)/HER1, HER2, and HER4.^14^ The pre‐clinical data confirmed the role of pyrotinib in irreversible inhibition of multiple ErbB receptors and the proliferation of HER2 overexpressing cells both in vivo and in vitro.^15^ Multiphase clinical trials on treatment of HER2‐positive metastatic breast cancer with pyrotinib have been performed. However, the effect of pyrotinib treatment on the final outcome of HER2‐positive breast cancer with brain metastasis was not fully elucidated. In this multicenter study, we try to study the efficacy and safety of pyrotinib on patients with HER2‐positive breast cancers showing brain metastasis.

## 2 MATERIALS AND METHODS

We recruited cases from 10 hospitals in China. An independent committee of radiologists from each participating hospitals was set up to retrospectively con‐firm the validity and the objective response rate (ORR, defined as the proportion of patients whose best overall response was either a complete or partial response), disease control rate (DCR) (defined as disease control rate was complete, partial response, and stable disease).

Investigations were performed in accordance with Chinese laws and regulations and the Helsinki declaration, after approval by the local ethics committee at each participating site.

### 2.1 Patient population

The HER2‐positive breast cancer with progressive brain metastasis at the time of pyrotinib initiation was included in the study.

The inclusion criteria: (1) The HER2‐positive breast cancer patients accompany with brain metastasis are confirmed by pathology and imaging studies, with a histologic or cytologic diagnosis of brain metastatic breast cancer (MBC); (2) HER2 is positive (3+ by Immunohistochemistry (IHC), or if 2+ by IHC, then confirmed by fluorescent in situ hybridization (FISH) with both gene amplification; (3) ECOG scoring 0–2; (4) previously received anti‐HER2 therapies or not; (5) The brain metastasis met progressive at the time of pyrotinib.

All the patients received oral pyrotinib 400mg once per day for 21 days as a cycle, and the patients were evaluated every two cycles. All the pertinent data including medical history, clinical examination, histopathology, immunohistochemistry, radiographic imaging, treatment outcome, and adverse reactions are recorded for final assessments. Patients treated with pyrotinib and other modalities were compared with those using pyrotinib alone. And for those patients using pyrotinib combined with chemotherapy but stopped due to intolerance within 15 days or termination of chemotherapy for other reasons are defined as pyrotinib alone.

In the intracranial radiotherapy group, individual radiotherapy plans were made in different centers according to the differences of patients’ conditions.

### 2.2 Study end points and assessments

The primary end point was the CNS and extra‐CNS ORR and DCR which were investigated according to the Response Evaluation Criteria in Solid Tumors RECIST Version 1.1, and are assessed by the investigators. Complete, partial response, and stable disease could be claimed only if confirmed by the first imaging examination.

The secondary end points included PFS, defined as the time of inclusion to the date of event to disease progression as assessed by the investigator according to RECIST, version 1.1, terminated due to unacceptable toxicity, or death.

Patients initially diagnosed as brain metastasis, with nausea, vomiting, headache, dizziness, and other intracranial symptoms would be improved after treatments. The proportion of patients with symptom relief was defined as the rate of brain symptom relief after treatments. Adverse events were compiled according to the National Cancer Institute Common Terminology Criteria for Adverse Events, version 4.0.

### 2.3 Statistical analyses

Statistical analysis was performed using Medcalc version 19.5.6. All *p* values were two‐sided, and those less than 0.05 were regarded as statistically significant. The survival data were estimated by the Kaplan–Meier method. The local control of brain metastasis was analyzed by the chi‐squared test.

## 3 RESULTS

### 3.1 Patient characteristics

Forty‐two female patients (median age, 51 years; range, 24–77 years) were enrolled from November 2018 to August 2019. The last patient has been enrolled for more than 18 months until the end of follow‐up. The baseline characteristics of the 42 patients are presented in Table 1. ECOG scoring of all patients was 0–2 point. Nineteen patients showing intracranial symptoms (nausea, vomiting, dizziness, etc.) were recorded.

**TABLE 1:** Patients’ demographic and baseline characteristics

Characteristics	No.	%
With other site metastasis		
Yes	38	90
No	4	10
Brain radiotherapy		
Yes	30	82
No	12	28
Combined chemotherapy		
Yes	25	59
No	17	41
Previously received anti‐HER2 therapy		
Yes	39	93
No	3	7
Symptomatic brain metastases		
Yes	19	45
No	23	55
With meningeal metastases		
Yes	4	5
No	38	95
Number of brain metastases		
≦5	35	83
>5	7	17

### 3.2 Efficacy

**FIGURE 1:**
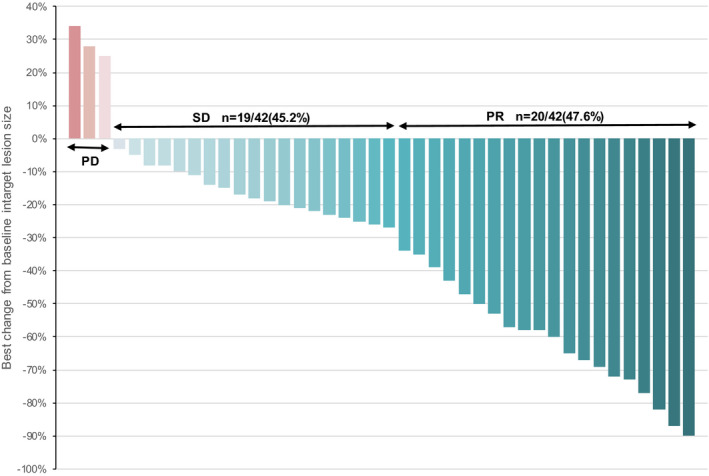
The figure of local control

The ORR and DCR of CNS were observed in 20 of 42 (47.6%) and in 39 of 42 (92.8%). The ORR and DCR of extra‐CNS were observed in 9 of 38 (23.6%) and in 36 of 38 (94.7%). The compounded ORR and DCR were observed in 17 of 42 (40.4%) and in 39 of 42 (92.8%) (Tables 2 and 3; Figure 1). There was no statistical significance between the ORR and DCR of CNS with radiotherapy and without radiotherapy (*p *= 0.426, 0.222, respectively). The improvement rate of craniocerebral symptoms after treatment was (19/19) 100%, with the median duration of response of 15 months. The median effective time of brain metastases and other metastases was 43 and 50 days. Effective time was defined as the first imaging evaluation and different image assessment methods may be applied in different centers.

**TABLE 2:** Parameters of different assessment

Parameters	No.	%
ORR of CNS	CR (0) + PR (20)	47.6
DCR of CNS	CR (0) + PR (20) + SD (19)	92.8
ORR of CNS with radiotherapy	CR (0) + PR (16)	55.1
DCR of CNS with radiotherapy	CR (0) + PR (16) + SD (12)	96.5
ORR of CNS without radiotherapy	CR (0) + PR (4)	30.7
DCR of CNS without radiotherapy	CR (0) + PR (4) + SD (7)	84.6
ORR of extra‐CNS	CR (0) + PR (9)	23.6
DCR of extra‐CNS	CR (0) + PR (9) + SD (27)	94.7
The compounded ORR	CR (0) + PR (17)	40.4
The compounded DCR	CR (0) + PR (17) + SD (22)	92.8

**TABLE 3:** Adverse reactions (AE)

AE	No.	%
Nausea and vomiting	1	2.4
Diarrhea	13	31
Grade 1	7	16.6
Grade 2	2	4.7
Grade 3	3	7.1
Grade 4	1	2.4
Hand foot syndrome	1	2.4
Myelosuppression	1	2.4

### 3.3 Survival analysis

The median follow‐up time was 22 months (interquartile range, 16.0–24.3 months). The number of events for PFS were 76.19%. In the 29 patients who progressed, the site of progression was CNS in two (6.9%) patients, extra‐CNS in 12 (41.3%) patients and both CNS and extra‐CNS lesions identified in 15 (51.7%) patients.

**FIGURE 2:**
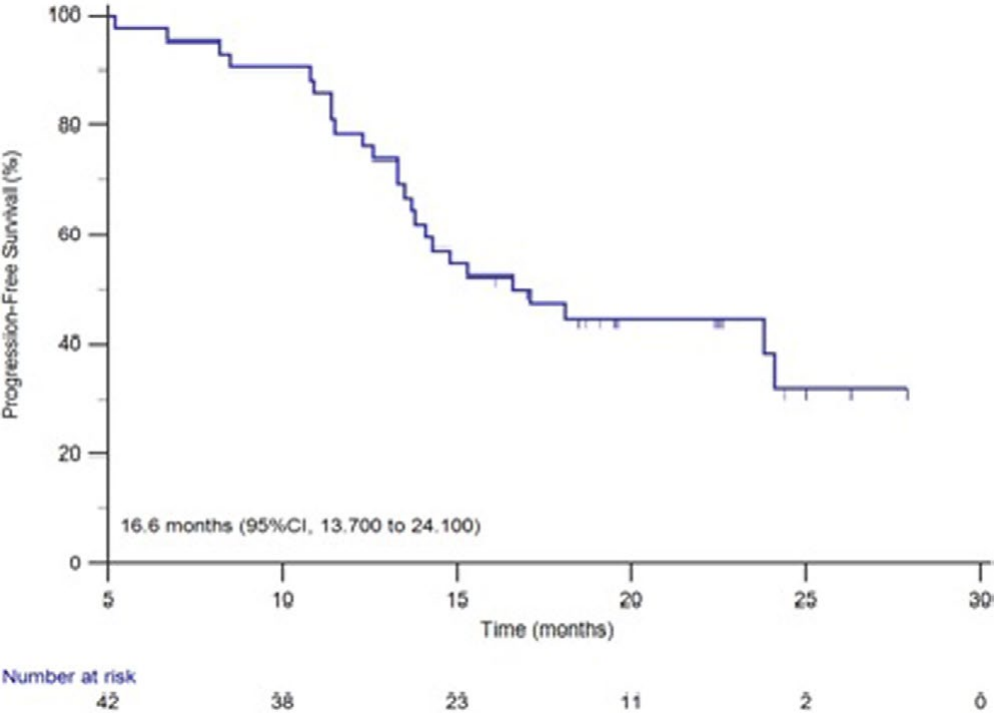
The survival curve of PFS of brain metastases

**FIGURE 3:**
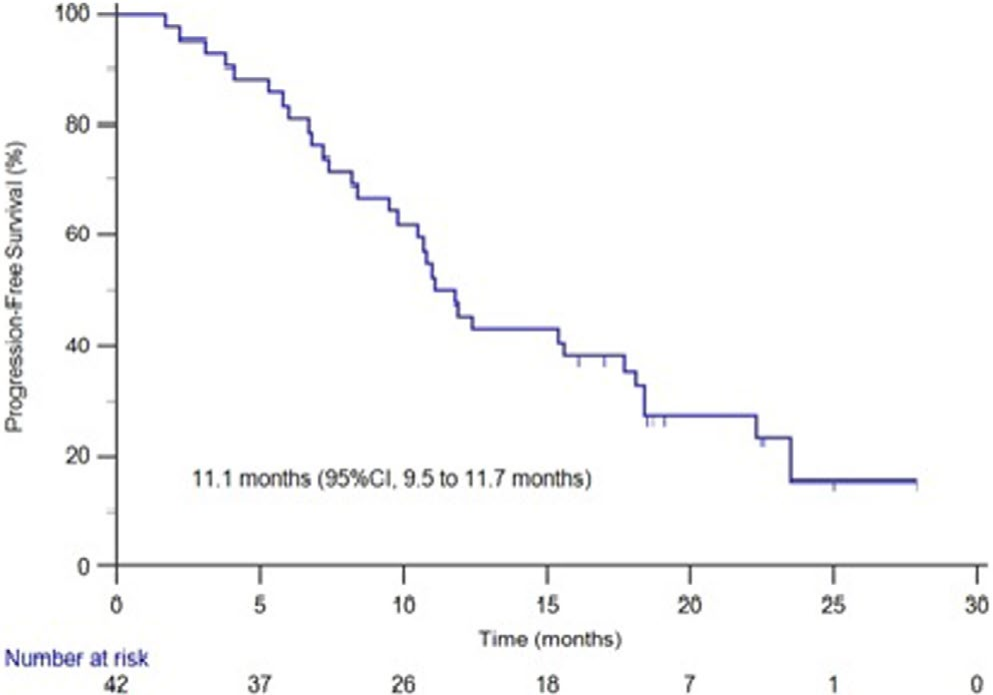
The survival curve of PFS in our group patients

**FIGURE 4:**
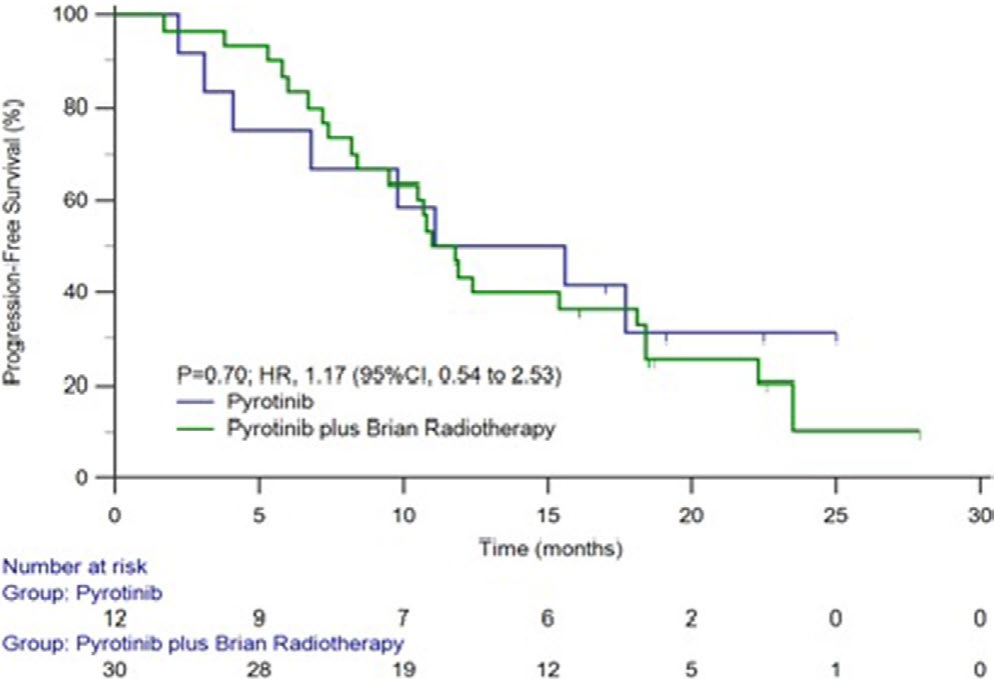
The survival curve of PFS with or without brain radiotherapy in our group patients

The median time for progression in brain metastasis and the study group patients was 16.6 and 11.1 months and the survival curve was shown in Figures 2 and 3. The median time for progression was 11.0 months in patients treated with pyrotinib combined with brain radiotherapy and 11.1 months in the patients with pyrotinib alone. No statistical significance was observed (*p* = 0.6985) (Figure 4).

**FIGURE 5:**
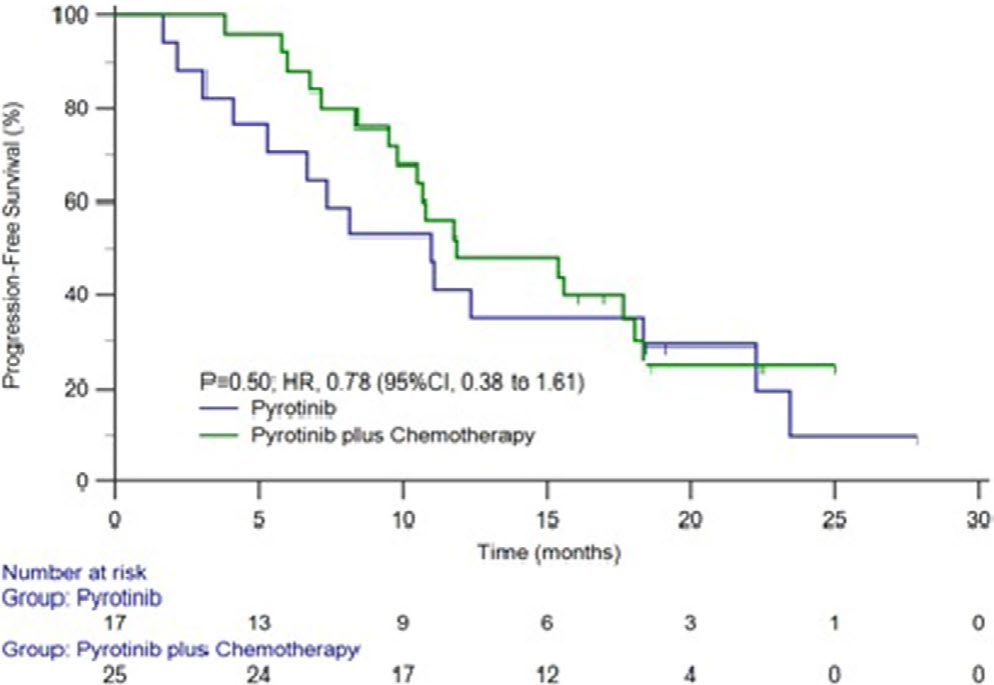
The survival curve of PFS combined and not combined chemotherapy with pyrotinib

The median time for progression was 11.9 and 11.0 months, respectively in patients received combined chemotherapy with pyrotinib and standalone pyrotinib therapy, which showed a trend for longer PFS with patient group receiving combined with chemotherapy, however, there was no statistical significance (*p *= 0.4989) (Figure 5). The OS for 24 months was 64.3%.

Among those 42 patients, 15 patients died, and 6 of them died of pulmonary metastasis, two died of brain metastasis, one died of liver metastasis, and the rest of patient have causes of death unknown listed and the OS data are immature.

### 3.4 Safety

Sixteen patients (36%) with adverse effects were recorded in the study, one with vomiting, 13 with diarrhea (seven with grade 1 diarrhea, two with grade 2 diarrhea, three with grade 3 diarrhea, one with grade 4 diarrhea), one with hand foot syndrome, one with myelosuppression, shortening study due to adverse effects in five cases, only one with termination of medication usage due to severe diarrhea. Toxicity‐related treatment discontinuations were recorded only in one patient. Reduced dose of pyrotinib in five patients was due to diarrhea. (Table 2).

## 4 DISCUSSION

The overexpression of HER2 was historically associated with poor prognosis in breast cancer.^16^, ^17^, ^18^, ^19^ Clinically, various targeted therapies and clinical trials have been reported in recent years to improve the outcomes of HER2‐positive breast cancers. In most reported studies, anti‐HER2 therapy was reported to delay the occurrence of brain metastasis.^12^, ^13^ But for those HER2‐positive breast cancer patients already with brain metastasis, the clinical management still remains a challenge.

The phase I study to evaluate the safety, and tolerability of pyrotinib in patients with HER2‐positive metastatic breast cancer was done in 2017 and showed significant improvement in response rate and PFS with good tolerance.^20^ In August 2018, pyrotinib was approved by China FDA to be used in combination with capecitabine for the treatment of HER2‐positive, advanced or metastatic breast cancer. In the latest phase II multicenter study,^21^ the combined therapy of pyrotinib and capecitabine resulted significantly better overall response rate and PFS than lapatinib plus capecitabine in female HER2‐positive metastatic breast cancer previously treated with other modalities. However, this study did not investigate the effect of pyrotinib in HER2‐positive breast cancer patients with brain metastasis, and the efficacy and safety of pyrotinib in patients with brain metastasis are not yet clearly elucidated.

Javier Corte et al. reported that HER2‐positive breast cancer patients whose disease progressed during prior trastuzumab‐based therapy, the ORR and DCR were 3.4% and 10.3%, respectively, during pertuzumab monotherapy, with the addition of trastuzumab, the ORR and DCR were 17.6% and 41.2%, respectively.^22^ The local control rate of reorganized patients was relatively low, which may be related to the fact that there were many metastasis sites in the group of patients. While Fei Ma et al., reported that the ORR was 78.5% with pyrotinib and capecitabine and 57.1% with lapatinib and capecitabine.^20^ The ORR of brain metastasis with lapatinib in the treatment of HER2‐positive brain metastasis was 65.9%.^23^ In the treatment of HER2‐positive brain metastases with lenvatinib alone, the ORR was not significant. A meaningful ORR (49%) can be obtained in combination with capecitabine.^24^ The efficacy of radiotherapy‐based approaches was largely unknown in the specific setting of brain metastases from HER2‐positive breast cancer.

In this study, we retrospectively analyzed a series of 42 HER2‐positive breast cancers with brain metastasis treated with pyrotinib. The study revealed that the ORR and DCR of CNS were 47.6% and 92.8%, respectively. The ORR and DCR of extra‐CNS were 23.6% and 94.7%, respectively. The local control rate was consistent with Ma et al. study and comparatively better than other reports, highlighting the role of pyrotinib in the possible management in those patients. Furthermore, the remission rate of craniocerebral symptoms is as high as 100% in HER2‐positive breast cancer with brain metastasis treated with pyrotinib, which significantly improved the life quality of those patients. The local control rate of brain metastasis treated with pyrotinib and radiotherapy was higher than those without radiotherapy, but there was no statistical significance, which may be due to the small molecular weight of pyrotinib and easy to enter the blood–brain barrier. Therefore, for HER2‐positive breast cancer patients with brain metastases, if the burden of brain metastases is small and the symptoms are mild, pyrotinib can be used alone and the financial burden of patients can be reduced.

The median time for progression in our group patients was 11.1 months which is higher than 5.5 months of lapatinib plus capecitabine with brain metastases, but is lower than 18.1 months of pyrotinib combined with capecitabine which was not analyzed in patients with brain metastasis. As some of the patients in this study were intolerant with chemotherapy, they only received pyrotinib monotherapy. In this study, most patients have other organ metastasis and been receiving multiple treatment modalities. Our study showed that the median PFS was 11.0 months in patients with pyrotinib and brain radiotherapy, and 11.1 month in the patients treated with only pyrotinib which shows no statistical significance. The median PFS in patients treated with pyrotinib and chemotherapy (11.9 months) was longer than single use of pyrotinib (11.0 months), also without a statistical significance. Therefore, for HER2‐positive breast cancer patients with brain metastases, the use of pyrotinib alone without the combination of brain radiotherapy, was also beneficial. The initial benefit of pyrotinib in combination with chemotherapy was not statistically significant compared with pyrotinib alone, probably because our group of patients, with a relatively short follow‐up period, had not all yet reached the end of the event. In another just published study,^25^ the median PFS and OS were 8.67 and 13.93 months, respectively in the 39 HER2‐positive breast cancer patients with brain metastases, and they also confirmed the prolonged survival of combined therapy of pyrotinib with surgery or radiation in these patients.

Recently, tucatinib, the new generation TKI, had also been studied to improve the antitumor activity against brain metastases in patients with HER2‐positive breast cancer.^26^

Diarrhea is the most common adverse effect observed with tyrosine kinase inhibitors targeting epidermal growth factor receptor/HER2. In this study group, 36% patients with adverse effects were recorded, 31% with diarrhea, but only one case dropped out the study due to grade 4 diarrhea, the other side effects were all grade 1. No cases of toxic death were recorded. The incidence of adverse events we followed was lower than that reported in Ma et al. study,^21^ though slightly different, it is all manageable and safe.

Our study had several limitations. First, as it is a multicenter retrospective study, inevitable deviation may occur. Second, we did not measure pyrotinib concentrations in the cerebrospinal fluid, and therefore could not assess the pyrotinib penetration rate of the blood–brain barrier. Third, there was no OS data due to short follow‐up time. And finally, we did not assess the quality of life or the effect of treatment on the neurocognitive functions.

## 5 CONCLUSION

Currently, there is less data of pyrotinib in treatment HER2‐positive breast cancer patients with brain metastases, and the efficacy and safety of combined radiotherapy are unknown. This study show that pyrotinib alone led to significantly greater local control rates and PFS, with manageable toxicity for patients with HER2‐positive breast cancer with brain metastases. Further follow‐up study will provide more OS data.

## 6 Funding statement

The study was supported by Clinical Medical Research Center of Radiotherapy of Cancer of Shandong Province (2019CYXZX01).

## CONFLICT OF INTEREST

There is no conflict of interest to declare.

## AUTHOR CONTRIBUTIONS

Dr. Xiangjiao Meng and Chunjian Wang designed the study, analyzed the data collected, and revised the manuscript. Dr. Min Gao was responsible for all the data collection, data analysis, and manuscript preparing. The other authors collected the relevant data in their hospitals.

## Data Availability

The data that support the findings of this study are available upon request from the corresponding author. The data are not publicly available due to privacy or ethical restrictions.
